# Automated Diagnosis of Hepatitis B Using Multilayer Mamdani Fuzzy Inference System

**DOI:** 10.1155/2019/6361318

**Published:** 2019-02-05

**Authors:** Gulzar Ahmad, Muhammad Adnan Khan, Sagheer Abbas, Atifa Athar, Bilal Shoaib Khan, Muhammad Shoukat Aslam

**Affiliations:** ^1^Department of Computer Science, National College of Business Administration and Economics, Lahore, Pakistan; ^2^Department of Computer Science, CUI, Lahore, Pakistan; ^3^Department of Computer Science, Minhaj University, Lahore, Pakistan

## Abstract

In this research, a new multilayered mamdani fuzzy inference system (Ml-MFIS) is proposed to diagnose hepatitis B. The proposed automated diagnosis of hepatitis B using multilayer mamdani fuzzy inference system (ADHB-ML-MFIS) expert system can classify the different stages of hepatitis B such as no hepatitis, acute HBV, or chronic HBV. The expert system has two input variables at layer I and seven input variables at layer II. At layer I, input variables are ALT and AST that detect the output condition of the liver to be normal or to have hepatitis or infection and/or other problems. The further input variables at layer II are HBsAg, anti-HBsAg, anti-HBcAg, anti-HBcAg-IgM, HBeAg, anti-HBeAg, and HBV-DNA that determine the output condition of hepatitis such as no hepatitis, acute hepatitis, or chronic hepatitis and other reasons that arise due to enzyme vaccination or due to previous hepatitis infection. This paper presents an analysis of the results accurately using the proposed ADHB-ML-MFIS expert system to model the complex hepatitis B processes with the medical expert opinion that is collected from the Pathology Department of Shalamar Hospital, Lahore, Pakistan. The overall accuracy of the proposed ADHB-ML-MFIS expert system is 92.2%.

## 1. Introduction

Disease analysis is a crucial element in the field of medicine and healthcare. An inappropriate analysis of a disease often results in improper treatment that leads to complications of the ailment and eventually to death [1]. What are the major signs and symptoms of the disease and its extent or degree of symptoms on the organs? When this is resolved, suitable treatment can be administered to lighten the pains. To perform this efficiently at the right time is complicated and needs much knowledge about the disease and history of the patient. It is essential to analyze the disease at the right time and report its conditions. As hepatitis is a liver infection disease, it may cause death if not diagnosed at the right time. These are various symptoms for an abnormal liver. The cause of hepatitis B includes the use of addictive drugs, continuous use of alcohol and medicines, smoking, sharing of daily use utensils with an infected person, blood transfusion, sexual contact with infected person, etc. It is common in areas where the system of sanitation is absent and blood transfusion without proper protection is being performed [2]. Many approaches for analysis have been explored. Some of those are crucial physical examination, liver tests, ultrasound, liver biopsy, blood tests, etc. Different blood tests are conducted for hepatitis B. After the test of ALT [13] and AST, the major test is hepatitis B surface antigen (HBsAg) [12, 18]. If the HBsAg test result is positive, then other tests such as anti-HBsAg, anti-HBcAg, anti-HBcAg-IgM, HBeAg, anti-HBeAg, and HBV-DNA [17] must be conducted to check the level of hepatitis. If chronic hepatitis is severe, it causes health issues. It can be classified into five phases: (i) HBeAg-positive chronic infection, (ii) HBeAg-positive chronic hepatitis, (iii) HBeAg-negative chronic infection, (iv) HBeAg-negative chronic hepatitis, and (v) HBsAg-negative phase [13]. Hepatitis-B virus (HBV) infection is still a problem for global public health with substantial morbidity and mortality [13–16]. If HBsAg is negative, then there are very fewer chances of HBV. Sometimes HBsAg is negative and anti-HBsAg (HBsAb) values are more than the cutoff values due to some previous vaccination. This results in no-hepatitis B state. In anti-HBcAg, anti-HBsAg is positive with negative HBsAg which is due to the previous recovered HBV attack. For acute hepatitis B, the HBsAg and anti-HBcAg-IgM must be positive. If the test results of HBsAg and anti-HBcAg are positive and anti-HBsAg and anti-HBcAg-IgM are negative, it results in chronic hepatitis B. The proposed ADHB-ML-MFIS expert system is based on these test results. There are different data analysis techniques, and some of them are based on machine learning, statistics, data abstraction, decision support system, and expert system [3]. Expert system techniques have been used in last few years in medical analysis. They increase the diagnostic accuracy and decrease the costs [4].

In all over the world, last-stage liver infection is a major source of morbidity and death [17]. In 2015, according to the World Health Organization (WHO), 1.34 million deaths were occurred due to hepatitis and 257 million people were infected with HBV worldwide [18]. In 2016, the WHO reported that approximately 240 million people had chronic hepatitis B virus infection from all over the world [19].

At present, artificial intelligence is being used to diagnose different kinds of medical problems. Intelligent systems are being developed to resolve the medicals issues [5]. Fuzzy inference system is the very powerful expert system to analyze the problems and provide their solutions. FIS is very useful where chances of uncertainty may occur. It is used in every filed of life such as automatic robotics, industries, computer sciences, medical systems, weather forecasting, agriculture, and so on. Neshat et al. presented a fuzzy system for the analysis and diagnosis of liver disorders [4]. Obot and Udoh diagnosed hepatitis using the fuzzy inference system on the basis of symptoms such as vomiting, body weakness, nausea, bile in urine, loss of appetite, jaundice, etc. [6]. Lancaster introduced a medical device on the basis of fuzzy logic control (FLC). FLC is used for managing the controller that employs air stress of human skin, and to manage it, alarm was used [7]. Rana and Sedamkar designed an expert system for medical diagnosis using the fuzzy logic inference system [8]. Adeli et al. discussed and diagnosed hepatitis in their research. They introduced “New Hybrid Hepatitis Diagnosis System Based on Genetic Algorithm and Adaptive Network Fuzzy Inference System” [9]. Dagar et al. introduced a FIS to diagnose various diseases based on initial symptoms [10]. Umoh and Ntekop proposed an expert system using the FIS to diagnose and monitor cholera [11].

## 2. Methods

**Figure 1:**
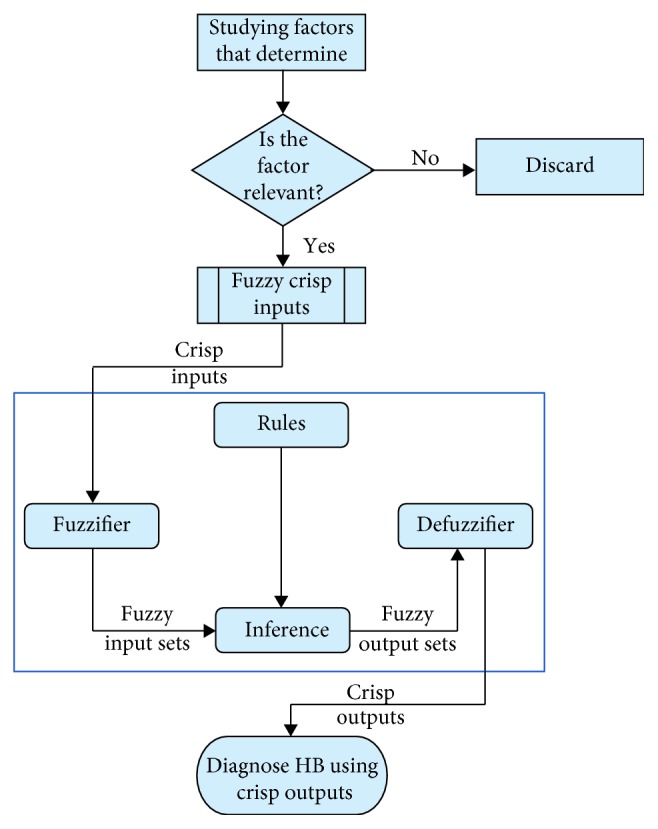
Proposed ADHB-MFIS-based expert system methodology.

Our proposed automated diagnosis hepatitis B (ADHB) multilayered mamdani fuzzy inference system- (MFIS-) based expert system (ADHB-ML-MFIS ES) is explained in this section.  Figure 1 shows the flow of the proposed ADHB-ML-MFIS expert system methodology.

**Figure 2:**
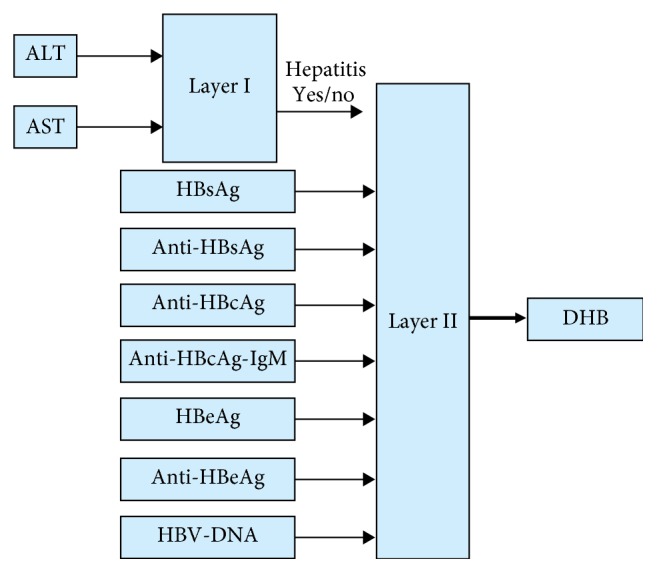
Proposed ADHB-ML-MFIS expert system.

The ADHB-ML-MFIS expert system consists of two layers as shown in Figure 2. In layer I, hepatitis is diagnosed (No/Yes) using two input variables, alanine aminotransferase (ALT) and aspartate aminotransferase (AST), as shown in Figure 2.

The value of ALT and AST are also used to build up a lookup table given in Table 1 to evaluate the status of hepatitis. If layer I diagnoses hepatitis, then layer II is active. Layer II diagnoses the stage of HB based on the seven input variables as shown in Figure 2. Layer II input variables are shown in Table 2.

The layer I of the proposed ADHB-ML-MFIS expert system can be mathematically written as

(1) $$\begin{array}{c} \mu_{\text{DH,layer}1}=\text{MFIS}\left[\mu_{\text{ALT}},\mu_{\text{AST}}\right], \end{array}$$

and the layer II of the proposed ADHB-ML-MFIS expert system can be expressed as

(2) $$\begin{array}{c} \mu_{\text{DHB, layer}2}=\text{MFIS }\left[\begin{array}{c} \mu_{\text{DH,layer}1},\;\;\mu_{\text{HBsAg}},\;\;\mu_{\text{anti}-\text{HBsAg }},\mu_{\text{anti}-\text{HBcAg}},\\ \mu_{\text{anti}-\text{HBcAg}-\text{lgM}},\mu_{\text{HBeAg}},\;\;\mu_{\text{anti}-\text{HBeAg}},\mu_{\text{HBV}-\text{DNA}} \end{array}\right]. \end{array}$$

### 2.1. Input Variables

Fuzzy input variables are statistical values that are used to diagnose hepatitis B. In this search, a total of nine different types of input variables are used on both layers. Two variables are used at layer I, and rest of the variables are used at layer II. The details of these input variables with their ranges are shown in Tables 1 and 2.

### 2.2. Output Variables

In this search, multilayered architecture is proposed to diagnose hepatitis B. If the layer I output is yes, then layer II is activated. Output variables for both layers are shown in Table 3.

### 2.3. Membership Functions

The membership function of this system gives curve values between 0 and 1 and also provides a mathematical function that offers statistical values of input and output variables. Graphical and mathematical representations of the proposed ADHB-ML-MFIS expert system member functions of I/O variables of both layers are shown in Table 4. These MFs are developed after discussion with medical experts from Pathology Department, Shalamar Hospital, Lahore, Pakistan.

### 2.4. Lookup Table

The lookup table for the proposed ADHB-ML-MFIS-based expert system contains 50 input-output rules. A few of them are shown in Table 5. This lookup table is developed with the help of medical experts from Pathology Department of Shalamar Hospital, Lahore, Pakistan.

### 2.5. I/O Rules

**Figure 3:**
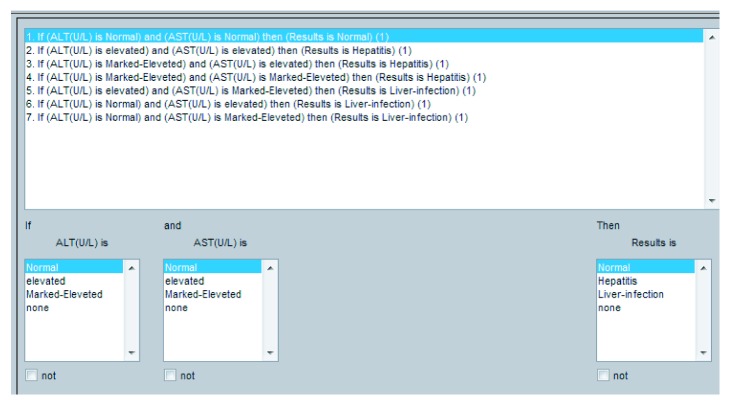
Layer I I/O rules for the proposed ADHB-ML-MFIS expert system.

**Figure 4:**
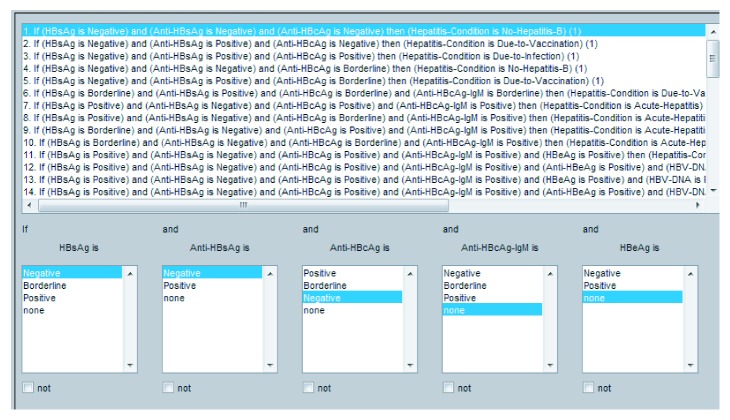
Layer II I/O rules for the proposed DHB-ML-MFIS expert system.

They play a critical role in any fuzzy inference system (FIS). The performance of any expert system depends upon these rules. In this research, I/O rules are developed using a lookup table as shown in Table 6. Proposed I/O rule based on the ADHB-ML-MFIS expert system is shown in Figures 3 and 4.

### 2.6. Inference Engine

Inference engine is one of the core components of any expert system. In this research, Mamdani inference engine is used in both layers.

### 2.7. Defuzzifier

**Figure 5:**
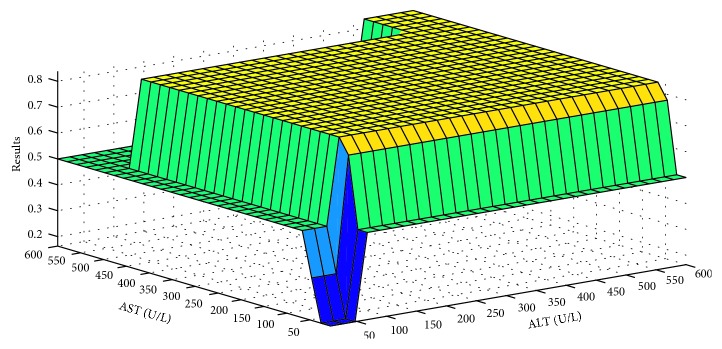
Layer I rules surface for ALT and AST.

**Figure 6:**
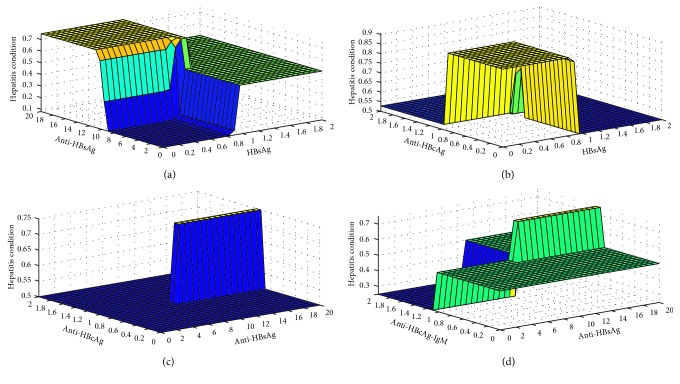
Rule surface for (a) anti-HBsAg and HBsAg, (b) anti-HBcAg and HBsAg, (c) anti-HBcAg and anti-HBsAg, and (d) anti-HBcAg-LGM and anti-HBsAg.

Defuzzifier is one of the critical components of an expert system. There are different types of defuzzifiers. In this research, a centroid-type defuzzifier is used.  Figure 5 shows the defuzzifier graphical representation of layer I in the ADHB-ML-MFIS expert system. In Figures 6(a)–6(d), the graphical representations of the defuzzifier at the layer II ADHB-ML-MFIS expert system is presented.

In Figure 5, diagnoses of hepatitis using probability are based on two input parameters ALT and AST. If the values of ALT and AST are elevated and ALT level is higher than the AST level, then there is 80% chance for hepatitis to occur. In this case, more than 80 % chances of hepatitis are present. Our system diagnoses hepatitis. It is also observed that if the AST level is higher than the ALT level, then there is fair chance for hepatitis to occur. If both values of ALT and AST are in the normal range, then it means no hepatitis.

Figure 6(a) shows hepatitis B (regarding probability) based on HBsAg and anti-HBsAg. Different colours in the surface region present the stages of hepatitis. It is also observed that if anti-HBsAg (*x*-axis) is negative (equivalent mathematically lies between 2 and 10 IU/L) and HBsAg (*y*-axis) is less than 0.8, then the probability of hepatitis B (*z*-axis) is 0; that is, it may be any other type of hepatitis. It is also observed that if costs of anti-HBsAg is more the 10 IU/L its mean positive, amounts of HBsAg is less the 0.8, and the value of hepatitis is 80% which is due to vaccination or some previous infection.

Similarly, remaining Figures 6(b)–6(d) present hepatitis B results by prevailing different input parameter values. The surface region represents probability values by two input variables from the given seven input variables. The hepatitis B results are the combination of at least three input variables.

## 3. Results

**Figure 7:**
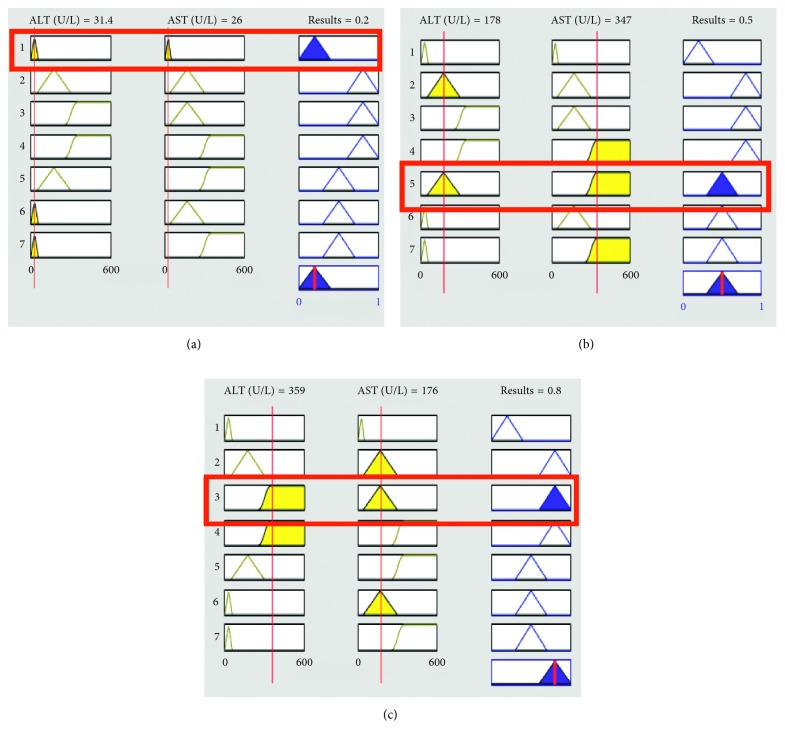
Layer I. Lookup diagram for (a) normal, (b) the other liver infections, and (c) hepatitis.

For simulation results, MATLAB R2017a tool is used. MATLAB is also used for modelling, simulation, algorithm development, prototyping, and many other fields. MATLAB is an efficient tool for programming, data analysis, visualization, and computing. For simulation results, nine inputs and one output DHB variables are used. When results of layer I show hepatitis, there can be different types of hepatitis such as hepatitis A, B, C, D, and E. In this research, the proposed ADHB-ML-MFIS-based expert system not only diagnosed hepatitis B but also showed the different levels of hepatitis B such as acute, chronic, etc. But if layer I diagnoses hepatitis and layer II diagnoses no hepatitis B, its means that it may contain other types of hepatitis. Figures 7(a)–7(c) show the performance of the proposed ADHB-ML-MFIS expert system at layer I.

Figure 7(a) shows that if the values ALT and AST are in the normal range, then there is no hepatitis or other infections.  Figure 7(b) shows that if the values of AST are greater than ALT, then the elevation may be due to alcohol or any other problem.  Figure 7(c) shows the high cost of ALT as it is more elevated than AST showing hepatitis.

Table 6 shows the accuracy of the proposed ADHB-ML-MFIS expert system in comparison with Medical Human expert of Shalamar Hospital, Lahore, Pakistan. The efficiency of the proposed method is randomly checked on 52 records. The standard unit of anti-HBsAg and HBV-DNA is IU/L; during simulation in most cases, we considered their values are 20 IU/L. The proposed DHB-ML-MFIS expert system provides the accurate results for all costs, and only at borderline it may achieve some minor errors.

**Figure 8:**
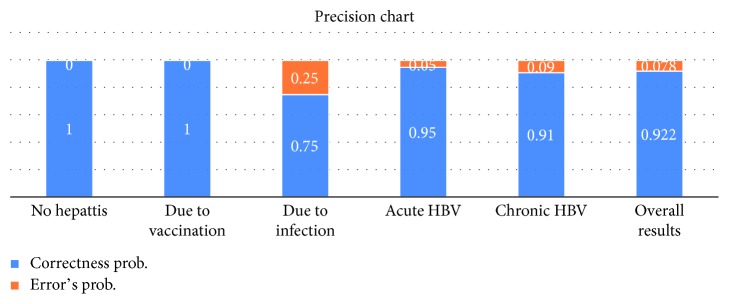
Precision of the proposed ADHB-ML-MFIS expert system.

Figure 8 shows the precision of the proposed ADHB-ML-MFIS expert system in the form of probability of all output cases. The last column produces an overall efficiency of the proposed ADHB-ML-MFIS expert system which is 92.2%.

## 4. Conclusion and Future Work

The primary focus of our research was to design an expert system to diagnose hepatitis B by ELISA blood test reports taken from Pathology Department of Shalamar Hospital, Lahore, Pakistan. The proposed expert system is elementary and easy to use for both medical and nonmedical professionals. A common man can also diagnose the status of hepatitis by providing required inputs. The primary objective of this research is to diagnose the different levels of hepatitis B. The overall precision of the proposed DHB-ML-MFIS expert system is 92.2%. In future, the efficiency of the proposed system can be improved using other techniques including computational intelligence such as neural network and neurofuzzy systems. This research work can be extended to others types of hepatitis such as A, C, D, and E.

## Figures and Tables

**Table 1:** Layer I input variables of the proposed ADHB-ML-MFIS expert system.

Sr no.	Input parameters	Ranges	Semantic sign	Reference range/cutoff value
1	AST	B/W 5–45 U/L	Normal	5–40 U/L
		B/W 40–550 U/L	Elevated values	
		GT > 500	Marked elevations	

3	ALT	B/W 7–55 U/L	Normal	7–55 U/L
		LT < 500	Elevated values	
		GT > 500	Marked elevations	

LT = less than; GT = greater than; B/W = between; U/L = unit per liter.

**Table 2:** Layer II input variables of the proposed ADHB-ML-MFIS expert system [13].

Sr no.	Input parameters	Ranges	Semantic sign	Reference range/cutoff value
1	HBsAg	LT < 0.9	Negative	1.0
		B/W 0.9–1.0	Borderline	
		GT > 1.0	Positive	

2	Anti-HBsAg	2–10 IU/L	Negative	10 IU/L
		GT > 10	Positive	

3	Anti-HBcAg	LT < 1.0	Positive	1.0
		B/W 0.9–1.1	Borderline	
		GT > 1.0	Negative	

4	Anti-HBcAg-IgM	LT < 1.0	Negative	1.0
		B/W 0.9–1.1	Borderline	
		GT > 1.0	Positive	

5	HBeAg	LT < 0.67	Negative	0.67
		GT > 0.67	Positive	

6	Anti-HBeAg	LT < 0.75	Positive	0.75
		GT > 0.75	Negative	

7	HBV-DNA	LT < 10	Negative	10 IU/L
		GT > 10	Positive	

LT = less than; GT = greater than; B/W = between; IU/L = international unit per liter; anti-HBsAg = HBsAb; anti-HBcAg-IgM = HBcAb-IgM; anti-HBcAg = HBcAb; anti-HBeAg = anti-HBeAg.

**Table 3:** Layers I and II output variables of the proposed ML-MFIS-DHB expert system.

Sr no.		Output variables	Semantic sign
1	Layer I	Hepatitis	No
			Yes
			Liver infection

2	Layer II	DHB	No hepatitis B
			Acute hepatitis
			Chronic hepatitis
			Immunity due to vaccination
			Immunity due to the previous infection

**Table 4:** Input and output variables membership functions used in the proposed ADHB-ML-MFIS expert system.

Sr no.	Input variables	Membership function (MF)	Graphical representation of MF
1	HBsAg = *S*(*μ*_S_(*s*))	*μ*_S,N_(*s*)={max(min(1, 0.9 − *s*/0.1), 0)} *μ*_S,BL_(*s*)={max(min(*s* − 0.8/0.1, 1, 1.1 − *s*/0.1), 0)} *μ*_S,P_ (*s*)={max(min(*s* − 1/0.1, 1 ), 0)}	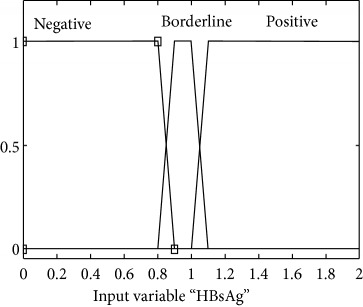

2	Anti-HBsAg = A (*μ*_A _(*a*))	*μ*_A,N_(*a*)={max(min(1, 10.5 − *a*/0.1 ), 0)} *μ*_A,P_(*a*)={max(min(*a* − 9.5/0.1, 1 ), 0)}	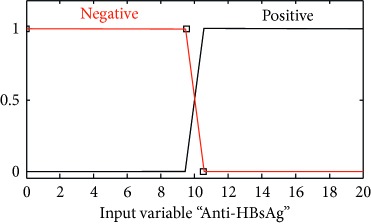

3	Anti-HBcAg = C (*μ*_C_(*c*))	*μ*_C,P_(*c*)={max(min(1, 0.95 − *c*/0.05), 0)} *μ*_C,BL_(c)={max(min(*c* − 0.9/0.05, 1, 1.1 − *c*/0.05), 0)} *μ*_C,N_(*c*)={max(min(*c* − 1.05/0.05, 1 ), 0)}	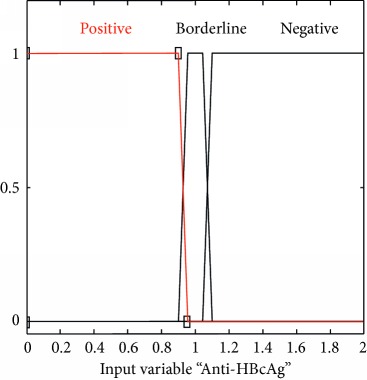

4	Anti-HBcAg-IgM = I (*μ*_*I*_(*i*))	*μ*_I,N_(*i*)={max(min(1, 0.95 − *i*/0.05), 0)} *μ*_I,BL_(*i*)={max(min(*i* − 0.9/0.05, 1, 1.1 − *i*/0.05), 0)} *μ*_I,N_(*i*)={max(min(*i* − 1.05/0.05, 1 ), 0)}	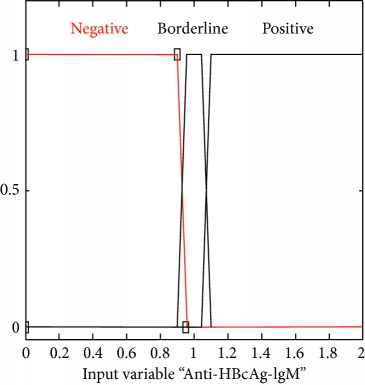
5	HBeAg = E (*μ*_E_(*e*))	*μ*_E,N_(*e*)={max(min(1, 0.69 − *e*/0.04), 0)} *μ*_E,P_(*e*)={max(min(*e* − 0.65/0.04, 1 ), 0)}	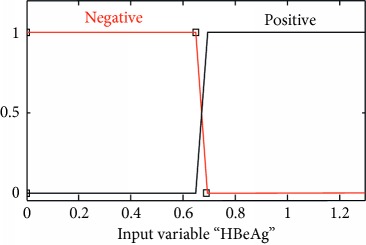

6	Anti-HBeAg = T (*μ*_*T*_(*t*))	*μ*_T,P_(*t*)={max(min(1, 0.8 − *t*/0.1), 0)} *μ*_T,N_(*t*)={max(min(*t* − 0.8/0.1, 1 ), 0)}	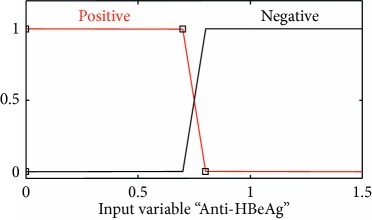

7	HBV-DNA = V (*μ*_V_(*v*))	*μ*_V,N_(*v*)={max(min(1, 10.5 − *v*/0.1), 0)} *μ*_V,P_(*v*)={max(min(*v* − 9.5/0.1, 1 ), 0)}	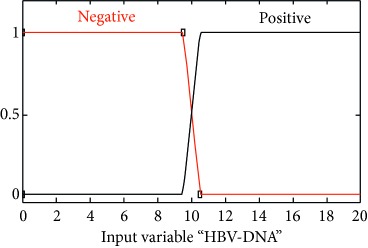

8	Hepatitis = H (*μ*_H_(*h*))	$\mu_{\text{H,N}}\left(h\right)=\left\{\begin{array}{c} 0.25-h/0.2,\;0\leq h\leq 0.25 \end{array}\right\}$ $\mu_{\text{H,A}}\left(h\right)=\left\{\begin{array}{cc} h/0.25, & 0\leq h\leq 0.25\\ 0.5-h/0.25, & 0.25\leq h\leq 0.5 \end{array}\right\}$ $\mu_{\text{H,C}}\left(h\right)=\;\left\{\begin{array}{cc} h-0.25/0.25, & 0.25\leq h\leq 0.5\\ 0.75-h/0.25, & 0.5\leq h\leq 0.75 \end{array}\right\}$ $\mu_{\text{H,V}}\left(h\right)=\;\left\{\begin{array}{cc} h-0.5/0.25, & 0.5\leq h\leq 0.75\\ 1-h/0.25, & 0.75\leq h\leq 1 \end{array}\right\}$ $\mu_{\text{H,I}}\left(h\right)=\;\left\{\begin{array}{c} h-0.75/0.25,\;0.75\leq h\leq 1 \end{array}\right\}$	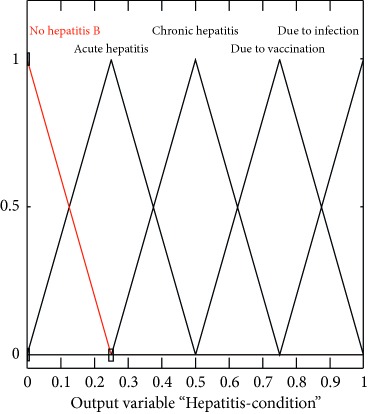

**Table 5:** Lookup table for the proposed ADHB-ML-MFIS.

Rules	HBsAg	Anti-HBsAg	Anti-HBcAg	Anti-HBcAg (IgM)	HBeAg	Anti-HBeAg	HBV-DNA	Results
1	N	N	N	—	—	—	—	None
2	N	P	N	—	—	—	—	Due to vaccination
3	N	P	P	—	—	—	—	Due to infection
4	P	N	P	P	—	—	—	Acute HBV
5	P	N	P	P	—	P	P	
6	P	N	P	P	P	—	P	
7	P	N	N	P	P	N	P	
8	P	N	P	P	P	N	N	
9	P	N	N	P	P	P	N	
10	P	N	P	N	—	—	—	Chronic HBV
11	P	N	P	N	—	—	P	
12	P	N	P	N	P	—	—	
13	P	N	P	N	P	—	—	
14	P	N	P	N	—	P	P	
15	P	N	P	N	P	—	P	

**Table 6:** Accuracy of the proposed ADHB-ML-MFIS expert system.

Patient	HBsAg	Anti-HBsAg	Anti-HBcAg	Anti-HBcAg (IgM)	HBeAg	Anti-HBeAg	HBV-DNA	Human expert decision	Proposed DHB-ML-MFIS expert system decision	Probability of correctness	Probability of errors
1	N (0.2)	N(7)	N(1.9)	—	—	—	—	None	None	1.0	0
2	N (0.4)	N(5)	BL(0.98)	—	—	—	—				
3	N (0.5)	P(20)	N(1.5)	—	—	—	—	Due to vaccination	Due to vaccination	1.0	0
4	N (0.7)	P(20)	BL(0.97)	—	—	—	—				
5	N (0.4)	P(20)	P(0.2)	—	—	—	—	Due to infection	Due to infection	0.75	0.25
6	BL (0.91)	P(20)	P(0.55)	—	—	—	—				
7	BL(0.91)	P(20)	P(0.37)	—	—	—	—				
8	BL(0.95)	P(20)	P(0.31)	—	—	—	—		**Chronic HBV**		
9	P (1.7)	N(3)	P(0.44)	P(1.67)	—	—	—	Acute HBV	Acute HBV	0.95	0.05
10	P(1.9)	N(5)	BL(0.98)	P(1.17)	—	—	—		Acute HBV		
11	BL(0.95)	N(7)	P(0.75)	P(1.31)	—	—	—		Acute HBV		
12	BL(0.95)	N(5)	BL(0.99)	P(1.57)	—	—	—		Acute HBV		
13	P(1.7)	N(6)	P(0.73)	P(1.43)	P(1.11)	—	—		Acute HBV		
14	P(1.48)	N(7)	P(0.48)	P(1.63)	—	P(0.56)	P(17)		Acute HBV		
15	P(1.45)	N(6)	P(0.25)	P(1.28)	P(0.87)	—	P(20)		Acute HBV		
16	P(1.65)	N(2)	P(0.45)	P(1.63)	—	N(1.23)	P(20)		Acute HBV		
17	P(1.4)	N(6)	P(0.7)	P(1.68)	P(0.9)	N(0.87)	—		Acute HBV		
18	P(1.31)	N(3)	P(0.55)	BL(0.99)	P(0.77)	P(0.45)	—		Acute HBV		
19	P(1.42)	N(6)	P(0.37)	BL(0.901)	P(0.8)	N(1.19)	—		**Chronic HBV**		
20	P(1.3)	N(4)	P(0.5)	BL(1.03)	P(0.85)	N(1.17)	P(20)		Acute HBV		
21	P(1.57)	N(3)	P(0.35)	BL(0.97)	P(0.97)	P(0.27)	P(20)		Acute HBV		
22	BL(0.91)	N(5)	P(0.47)	P(1.43)	P(0.80)	P(0.31)	P(20)		Acute HBV		
23	P(1.9)	N(4)	P(0.75)	P(1.29)	P(0.99)	P(0.65)	P(20)		Acute HBV		
24	BL(0.93)	N(8)	P(0.21)	P(1.65)	P(0.89)	N(1.05)	P(20)		Acute HBV		
25	P(1.8)	N(6)	BL(1.01)	P(1.43)	P(0.93)	N(1.17)	P(20)		Acute HBV		
26	P(1.31)	N(5)	N(1.7)	P(1.3)	P(0.87)	N(1.0)	P(20)		Acute HBV		
27	P(1.7)	N(8)	P(0.72)	P(1.29)	P(0.97)	N(0.87)	N(7)		Acute HBV		
28	P(1.4)	N(3)	N(1.38)	P(1.57)	P(0.73)	P(0.35)	N(5)		Acute HBV		
29	P(1.21)	N(6)	P(0.51)	P(1.81)	N(.35)	P(0.49)	N(7)		Acute HBV		
30	P(1.42)	N(5)	P(0.37)	N(0.41)	—	—	—	Chronic HBV	Chronic HBV	0.91	0.09
31	P(1.71)	N(7)	BL(0.93)	N(0.49)	—	—	—		Chronic HBV		
32	P(1.48)	N(2)	BL(1.08)	N(0.68)	—	—	P(20)		Chronic HBV		
33	P(1.2)	N(4)	P(0.2)	N(0.2)	—	—	P(20)		Chronic HBV		
34	P(1.7)	N(3)	P(0.25)	N(0.47)	P(1.2)	—	—		Chronic HBV		
35	P(1.3)	N(8)	P(0.65)	N(0.19)	P(0.92)	—	—		Chronic HBV		
36	P(1.5)	N(7)	P(0.72)	N(0.23)	—	P(0.37)	P(20)		Chronic HBV		
37	P(1.21)	N(3)	P(0.23)	N(0.51)	P(0.89)	—	P(20)		Chronic HBV		
38	P(1.35)	N(5)	BL(1.02)	N(0.45)	P(0.99)	—	P(20)		Chronic HBV		
39	P(1.5)	N(9)	P(0.72)	N(0.39)	P(1.0)	N(1.28)	—		Chronic HBV		
40	P(1.9)	N(4)	P(0.15)	N(0.23)	—	N(0.92)	P(20)		Chronic HBV		
41	P(1.4)	N(6)	BL(0.93)	N(0.76)	—	P(0.48)	P(20)		Chronic HBV		
42	BL(0.91)	N(7)	P(0.63)	N(0.45)	—	P(0.93)	P(20)		Chronic HBV		
43	BL(0.902)	N(3)	P(0.27)	N(0.23)	P(0.92)	—	P(20)		Chronic HBV		
44	BL(0.902)	N(5)	BL(1.02)	N(0.39)	P(0.82)	—	P(20)		**Acute HBV**		
45	P(1.2)	N(6)	BL(0.93)	N(0.18)	P(0.95)	N(1.4)	P(20)		Chronic HBV		
46	P(1.4)	N(6)	P(0.3)	N(0.47)	P(0.89)	N(1.15)	P(20)		Chronic HBV		
47	P(1.7)	N(8)	BL(0.93)	N(0.71)	N(0.45)	P(0.45)	N(5)		Chronic HBV		
48	P(1.3)	N(3)	P(0.85)	N(0.65)	N(0.32)	P(0.38)	N(4)		Chronic HBV		
49	P(1.7)	N(7)	BL(0.93)	N(0.42)	N(0.47)	P(0.52)	P(20)		Chronic HBV		
50	BL(0.93)	N(7)	P(0.86)	N(0.28)	N(0.31)	P(0.35)	P(20)		**No hepatitis**		
51	BL(.91)	N(4)	BL(0.96)	N(0.67)	N(0.37)	P(0.37)	P(20)		Chronic HBV		
52	P(1.3)	N(5)	P(0.25)	N(0.47)	N(0.46)	P(61)	P(20)		Chronic HBV		

## Data Availability

The clinical/patient data used to support the findings of this study are restricted by the Shalamar Hospital, Lahore, Pakistan, in order to maintain patient privacy. The simulation files/data used to support the findings of this study are available from the corresponding author upon request.

## References

[1] Ntaganda J. M., Gahamanyi M. (2015). Fuzzy logic approach for solving an optimal control problem of an uninfected hepatitis B virus dynamics. *Applied Mathematics*.

[2] Ejegwa P. A., Modom E. S. (2015). Diagnosis of viral hepatitis using new distance measure of intuitionistic fuzzy sets. *International Journal of Fuzzy Mathematical Archive*.

[3] Cheung N. (2001). Machine learning techniques for medical analysis.

[4] Neshat M., Yaghobi M. Designing a fuzzy expert system of diagnosing the hepatitis B intensity rate and comparing it with adaptive neural network fuzzy system.

[5] Sardesai A., Sambarey P., Kharat V., Deshpande A. Fuzzy logic application in gynecology: a case study.

[6] Obot O. U., Udoh S. S. A framework for fuzzy diagnosis of hepatitis.

[7] Lancaster S. S. A fuzzy logic controller for the application of skin pressure.

[8] Rana M., Sedamkar R. R. (2013). Design of expert system for medical diagnosis using fuzzy logic. *International Journal of Scientific and Engineering Research*.

[9] Adeli M., Bigdeli N., Afshar K. New hybrid hepatitis diagnosis system based on Genetic algorithm and adaptive network fuzzy inference system.

[10] Dagar P., Jatain A., Gaur D. Medical diagnosis system using fuzzy logic toolbox.

[11] Umoh U. A., Ntekop M. M. (2013). A proposed fuzzy framework for cholera diagnosis and monitoring. *International Journal of Computer Applications*.

[12] Cornberg M., Wong V. W.-S., Locarnini S., Brunetto M., Janssen H. L. A., Chan H. L.-Y. (2017). The role of quantitative hepatitis B surface antigen revisited. *Journal of hepatology*.

[13] European Association for the Study of the Liver (2017). EASL 2017 Clinical Practice Guidelines on the management of hepatitis B virus infection. *Journal of hepatology*.

[14] Craxi A., Wedemeyer H., Bjoro K. (2011). European association for the study of the liver. EASL clinical practice guidelines: management of hepatitis C virus infection. *Journal of hepatology*.

[15] Schweitzer A., Horn J., Mikolajczyk R. T., Krause G., Ott J. J. (2015). Estimations of worldwide prevalence of chronic hepatitis B virus infection: a systematic review of data published between 1965 and 2013. *The Lancet*.

[16] Lozano R., Naghavi M., Foreman K. (2012). Global and regional mortality from 235 causes of death for 20 age groups in 1990 and 2010: a systematic analysis for the Global Burden of Disease Study 2010. *The lancet*.

[17] Perz J. F., Armstrong G. L., Farrington L. A., Hutin Y. J. F., Bell B. P. (2006). The contributions of hepatitis B virus and hepatitis C virus infections to cirrhosis and primary liver cancer worldwide. *Journal of hepatology*.

[18] World Health Organization (2017). *Global Hepatitis Report 2017*.

[19] World Health Organization (2016). *Global Health Sector Strategy on Viral Hepatitis 2016-2021. Towards Ending Viral Hepatitis*.

